# Impact of COVID-19 Pandemic on the Incidence, Prehospital Evaluation, and Presentation of Ischemic Stroke at a Nonurban Comprehensive Stroke Center

**DOI:** 10.1155/2021/6624231

**Published:** 2021-01-18

**Authors:** Cesar Velasco, Brandon Wattai, Scott Buchle, Alicia Richardson, Varun Padmanaban, Kathy J. Morrison, Raymond Reichwein, Ephraim Church, Scott D. Simon, Kevin M. Cockroft

**Affiliations:** ^1^Penn State Comprehensive Stroke Center, Penn State Health, Hershey, PA, USA; ^2^Life Lion EMS, Penn State Health, Hershey, PA, USA; ^3^Department of Neurosurgery, Penn State Health, Hershey, PA, USA; ^4^Department of Neurology, Penn State Health, Hershey, PA, USA

## Abstract

**Introduction:**

Many reports have described a decrease in the numbers of patients seeking medical attention for typical emergencies during the COVID-19 pandemic. These reports primarily relate to urban areas with widespread community transmission. The impact of COVID-19 on nonurban areas with minimal community transmission is less well understood.

**Methods:**

Using a prospectively maintained prehospital quality improvement database, we reviewed our hospital EMS transports with a diagnosis of stroke from January to April 2019 (baseline) and January to April 2020 (pandemic). We compared the volume of patients, transport/presentation times, severity of presenting symptoms, and final diagnosis.

**Results:**

In January, February, March, and April 2019, 10, 11, 17, and 19 patients, respectively, were transported in comparison to 19, 14, 10, and 8 during the same months in 2020. From January through April 2019, there was a 53% increase in transports, compared to a 42% decrease during the same months in 2020, constituting significantly different trend-line slopes (3.30; 95% CI 0.48–6.12 versus -3.70; 95% CI -5.76–-1.64, *p* = 0.001). Patient demographics, comorbidities, and symptom severity were mostly similar over the two time periods, and the number of patients with a final diagnosis of stroke was also similar. However, the median interval from EMS dispatch to ED arrival for patients with a final diagnosis of stroke was significantly longer in January to April 2020 (50 ± 11.7 min) compared to the same time period in 2019 (42 ± 8.2 min, *p* = 0.01). *Discussion/Conclusion*. Our data indicate a decrease in patient transport volumes and longer intervals to EMS activation for suspected stroke care. These results suggest that even in a nonurban location without widespread community transmission, patients may be delaying or avoiding care for severe illnesses such as stroke. Clinicians and public health officials should not ignore the potential impact of pandemic-like illnesses even in areas of relatively low disease prevalence.

## 1. Introduction

In early 2020, the COVID-19 pandemic paralyzed many urban health systems across the United States and the world. Reports suggested a drop in emergencies including heart attack and stroke were due to individuals choosing to stay at home until their symptoms worsened rather than risk hospital exposure to the coronavirus [1]. According to physicians at urban medical centers, emergency departments were seeing fifty percent of the usual number of stroke cases and reporting lower than usual census in the intensive care units that typically managed this population [2].

Unlike urban areas, the impact of COVID-19 on nonurban hospitals with minimal community transmission has been less well understood or investigated. However, the same stress of social distancing, isolation, and reluctance to seek care for a stroke may place nonurban patients at similar risk of poor outcomes. In this study, we examined the impact of the COVID-19 pandemic on acute stroke care at a nonurban medical center located in an area without widespread community transmission.

## 2. Methods

We retrospectively reviewed prospective data from our institution's prehospital quality improvement database and cross-matched it against data entered in the hospital's Get With The Guidelines (GWTG) Stroke Center database. Emergency Medical Systems (EMS) transports with a diagnosis of stroke were compared from January to April 2019 (baseline) and January to April 2020 (pandemic). Variables of interest from the prehospital database included the volume of patients, the interval time between last known well (LKW) and time of EMS dispatch, prehospital transport and transfer times, severity of presenting symptoms, and final clinical diagnosis. Demographic data, as well as initial stroke severity and clinical outcomes, were obtained from the institution's GWTG Stroke Center database.

This research was conducted ethically under accepted guidelines and was approved by our facility's Human Subjects Protection Office and Institutional Review Board (Study 00015437). Deidentified data used in this project will be made available to other investigators upon reasonable request to the corresponding author. This project was supported by departmental funds.

### 2.1. Statistical Analysis

Data were analyzed using GraphPad Prism (version 8.2.1; San Diego, CA). Univariate between-group comparisons were calculated using Fisher's exact test for categorical data and unpaired *t*-tests for interval data. The Mann–Whitney *U* test was used for nonparametric ordinal data (e.g., NIHSS) and nonparametric continuous data (e.g., EMS time parameters). ANCOVA testing was utilized to compare the slopes of the linear regression lines. A *p* value of <0.05 was considered significant.

## 3. Results

**Figure 1:**
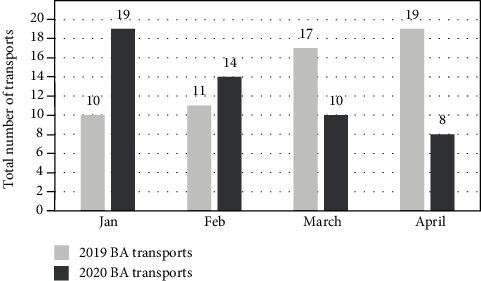
Total number of suspected stroke patients (aka brain attacks; BA) transported by EMS.

In the months of January, February, March, and April in 2020, the height of the COVID-19 pandemic in our region, 19, 14, 10, and 8 patients, respectively, were transported by our hospital EMS with a presumed diagnosis of stroke. In comparison, during the corresponding four months in 2019, 10, 11, 17, and 19 patients, respectively, were transported. This represents a 53% increase in transports for January through April of 2019, compared to a 42% decrease during the same months in 2020 (Figure 1). The slopes of these trend-lines are significantly different (3.30; 95% CI 0.48–6.12 versus -3.70; 95% CI -5.76–-1.64, *p* = 0.001). For the study period in 2019, 23 of 57 total patients transported were discharged with a final diagnosis of stroke compared to 21 of 51 patients in 2020. With the exception of a higher percentage of patients with diabetes in the pre-COVID 2019 period, there were no significant differences in demographics, comorbidities, or symptom severity between the two time segments for either the entire group of patients or those found to have a final diagnosis of stroke (Table 1). An examination of EMS time metrics found the median time on the scene for EMS was shorter in January through April of 2020 versus the same months in 2019 (14 + 5.9 versus 17 + 6.2 minutes, *p* = 0.028) (Table 2). However, despite this, the median interval from EMS dispatch to ED arrival for patients with a final diagnosis of stroke was significantly longer in January to April 2020 (50 ± 11.7 min) compared to the same time period in 2019 (42 ± 8.2 min, *p* = 0.01) (Table 2). For the most part, patients from the two time periods were similar in the initial stroke evaluation, acute management, and outcomes (Table 3). Patients in pre-COVID time period were more likely to have a positive EMS stroke screening tool, but no differences were seen in the location from which patients came or in their presenting NIHSS. A similar percentage of patients received IV thrombolysis, and door to needle times were similar. Short-term outcomes were also similar in terms of discharge NIHSS and discharge disposition.

## 4. Discussion

We examined the impact of the COVID-19 pandemic on our nonurban population. Prior publications have mostly focused on the impact of the pandemic on large urban centers where disease prevalence is relatively high and community transmission tends to be more widespread [3–6]. In the 12-county region of south-central Pennsylvania where our medical center is located, there were 335 COVID-19 cases in March and 4,652 in April (https://www.health.pa.gov/topics/disease/coronavirus/Pages/March-Archive.aspx, https://www.health.pa.gov/topics/disease/coronavirus/Pages/April-Archive.aspx) out of a population of approximately 2.1 million (approximately 10 to 221 per 100,000). In comparison, in the five-county Philadelphia metropolitan area with a population of approximately 4 million, there were 2,550 and 24,545 cases during the same two months, respectively (approximately 64 to 614 per 100,000) (https://www.health.pa.gov/topics/disease/coronavirus/Pages/March-Archive.aspx, https://www.health.pa.gov/topics/disease/coronavirus/Pages/April-Archive.aspx). In April, in New York City, one of the worst-hit areas in the United States, it was estimated by state officials that just over one-fifth of its residents had contracted COVID-19 [7]. Despite the relatively low disease prevalence in south-central Pennsylvania, we were concerned that people were inappropriately delaying emergency care out of fear of contracting the virus. Our present work appears to confirm this hypothesis. We found a significant decrease in the number of suspected stroke patient transports between January and April 2020, the peak of the pandemic in our region, versus the same months in 2019. These results suggest that even in nonurban areas with relatively low disease prevalence and community transmission, patients may be experiencing the same reticence in coming to the hospital. Unsurprisingly, given the widespread dissemination of COVID-19 information, the same fears and concerns present in areas of widespread community prevalence and transmission are also likely to be present in areas where infections are less common. Interestingly, there was no difference in the stroke severity at initial presentation or the rate of initial treatment with an intravenous thrombolytic. However, there was a trend toward more patients having a positive result on an EMS stroke screening tool in 2019 compared to 2020. Our examination of EMS time metrics indicated that overall time from dispatch to ED was increased in the first few months of 2020 compared to 2019, despite a slight decrease in the time on-scene. With the widely reported lack of traffic at this time, the reasons for this increase are unclear. Perhaps, concerns over appropriate personal protective equipment (PPE) may have led to some delays. Fortunately, discharge outcomes were similar, although the study was not powered to examine clinical outcomes.

The overwhelming fear of contracting COVID-19 may lead many patients to be reluctant to seek timely assistance for life-threatening emergencies, such as stroke [2, 8]. Although the practices of social distancing, sheltering in place, and self-quarantine are deemed effective in limiting the spread of the virus, these measures may also serve to discourage the pursuit of acute medical attention for non-COVID-related diseases [9]. Patient anxiety, depression, and concern for individual safety have all been cited as potential reasons for not seeking timely treatment [9]. Additionally, the observance of stay-at-home orders may have decreased the opportunity for friends and or family members to witness a patient exhibiting stroke symptoms in a timely manner [5]. Obviously, seeking immediate evaluation and treatment for stroke is imperative since the lasting consequences of delay can include long-term disability or death [10]. This study serves to reinforce the importance of stroke awareness education in the setting of a pandemic, even in areas where the pandemic may not have a profound, direct clinical impact.

Limitations to this study include the use of data associated with a single comprehensive stroke center; this is reflected in the relatively small number of suspected stroke cases analyzed between both time periods. Although data was prospectively acquired, this work involved a retrospective review and therefore is subject to the inherent biases of such studies. Although considered “nonurban” with significant rural areas, south-central Pennsylvania does include multiple towns and small cities where disease prevalence and community transmission rates may vary.

## 5. Conclusion

Our research suggests that nonurban communities with a low prevalence of COVID-19 and experiencing minimal community transmission would benefit from more public health education on the importance of seeking immediate care for acute stroke. Helping patients better understand the risks associated with avoiding medical treatment during a pandemic may help reduce the long-term financial and societal burden of stroke. As many areas prepare for second and third waves of infection, clinicians and public health officials need to be cognizant of the potential impact of the COVID-19 pandemic and other pandemic-like diseases on emergency care, even in areas where the direct effects of the pandemic may be less severe.

## Figures and Tables

**Table 1:** Demographics of all patients and patients with a final diagnosis of stroke.

	All patients			Stroke patients		
	2019 (pre-COVID-19)	2020 (COVID-19 era)	*p* value	2019 (pre-COVID-19)	2020 (COVID-19 era)	*p* value
Age (SD)	75.96 (13.2)	73.24 (17.3)	0.36	77.91 (12.6)	75.90 (18.9)	0.68
Female	*N* (63.2%)	*N* (56.9%)	0.56	56.5%	43.5%	0.55
Prior stroke/TIA				41.7%	58.3%	0.48
Hypertension				79.0%	76.5%	>0.99
Afib/flutter				15.8%	29.4%	0.43
CAD/prior MI				36.8%	17.6%	0.27
Carotid stenosis				0	0	>0.99
Diabetes mellitus				47.4%	11.8%	0.03
Tobacco abuse				15.8%	5.9%	0.61
Drug/alcohol abuse				10.5%	0%	0.49
Dyslipidemia				73.7%	58.8%	0.48
Family history of stroke				5.3%	23.5%	0.17

Demographic characteristics of patients pre- and post-COVID era presented as means and standard deviation for interval data and proportions for categorical data. Statistical significance for univariate between-group comparisons was calculated using Fisher's exact test for categorical data and unpaired *t*-tests for interval data.

**Table 2:** EMS time parameters for all patients and patients with a final diagnosis of stroke.

	All patients			Stroke patients		
	2019 (pre-COVID-19)	2020 (COVID-19 era)	*p* value	2019 (pre-COVID-19)	2020 (COVID-19 era)	*p* value
LKW to dispatch (median, IQR)	67 (19–347)	126 (12–310)	*p* = 0.86	70 (23–441)	126 (11.5–291.5)	*p* = 0.94
Time on scene (median, IQR)	16 (11.5–21)	14 (10–17)	*p* < 0.04	17 (11.5–21.25)	14 (13–17.5)	*p* = 0.48
Dispatch to ED (median, IQR)	45 (34–50.5)	45 (39–54)	*p* = 0.19	44.5 (34–49)	52 (42–59)	*p* < 0.03

All times in minutes. LKW: last known well; ED: emergency department. Mann–Whitney U test was used to compare EMS time parameters.

**Table 3:** Stroke evaluation, management, and outcome.

	2019 (pre-COVID-19)	2020 (COVID-19 era)	*p* value
Presenting location			
Residence	27	27	
Business	10	4	0.24
Healthcare	19	18	
Facility	1	4	
Other			
Positive EMS stroke scale	33/57 (57.9%)	19/51 (37.2%)	0.05
NIHSS on admission (median, range)	4 (0-25)	3 (0-24)	0.59
IV thrombolytic in ED	5/22 (22.7%)	4/21 (19.0%)	0.99
Door to needle (minutes)^†^	40.8	51.8	0.52
Stroke diagnosis confirmed	22/57	21/51	0.84
Stroke type			
Ischemic	15	10	
TIA	3	6	0.20
Hemorrhagic	4	5	
NIHSS at discharge (median, range)	3 (0-36)	1 (0-30)	0.43
Discharge disposition			
Home/rehab	44	35	0.39
Other	13	16	

EMS: Emergency Medical Services; NIHSS: National Institutes of Health Stroke Scale; IV: intravenous; ED: emergency department; TIA: transient ischemic attack. ^†^Reported only for those patients who received IV thrombolytics. Chi-square or Fisher's exact test for categorical variables. ^∗^Chi-square test for each variable within the EMS Stroke Scale. Mann–Whitney *U* test for nonparametric ordinal data (i.e., NIHSS).

## Data Availability

Deidentified data used in this project will be made available to other investigators upon reasonable request to the corresponding author.
